# The sequential mediating roles of alexithymia and interpersonal problems in the relationship between autistic traits and suicidal ideation: evidence from Chinese college students

**DOI:** 10.3389/fpubh.2025.1679206

**Published:** 2025-10-27

**Authors:** Sheliang Li, Wan Wang

**Affiliations:** ^1^School of Marxism, Henan Normal University, Xinxiang, China; ^2^Mental Health Education Center, Zhengzhou University, Zhengzhou, China

**Keywords:** suicidal ideation, autistic traits, alexithymia, interpersonal problems, Chinese

## Abstract

**Background:**

Although prior studies suggested that higher levels of autistic traits correlated with more suicidal thoughts and behavior, the specific underlying mechanism was not clear. The present study was designed to expand prior findings and test the mediating roles of alexithymia and interpersonal problems on the relation between autistic traits and suicidal ideation among large population-based college samples.

**Methods:**

A total of 6,763 college students (including 3,829 females) completed Autism-Spectrum Quotient, Toronto alexithymia Scale-20, Chinese Adolescents Self-Rating Life Events Checklist and Symptom Checklist.

**Results:**

1.54% college students reported high autistic traits and 9.54% college students had suicidal ideation. Autistic traits were positively correlated with suicidal ideation. The sequential mediating effects of alexithymia and interpersonal problems on the relation between autistic traits and suicidal ideation were significant.

**Conclusion:**

These findings contribute to our further understanding of how autistic traits affect suicidal ideation in the context of complex risks and outcomes. They are also helpful in the prevention and treatment of suicidal ideation and behaviors.

## 1 Introduction

The World Health Organization reported that nearly 1 million people die due to committing suicide each year all over the world. As a serious social and public health issue, suicide is one of the three top reasons of death in the 15–44 years age group, and the second main reason of death among the 15–19 years age group (1). Patton and his colleagues estimated that suicide will constitute 2.4% of the total disease burden by 2020, compared to 1.8% in 1998 (2). Suicidal ideation is the initial psychological activity of the suicide, and it refers to cogitation about the meaninglessness of life and death desires with specific suicide plans and excessive brooding with self-destruction (3). Suicidal ideation is a robust risk factor of suicide death (4), and it’s a complex phenomenon affected by social biological, individual, and environmental factors. Therefore, it is crucial to understand what increases the dangers of suicide and develops useful prevention, assessment, and therapy approaches.

Autism spectrum disorder (ASD) is a neurodevelopmental problem featured by social interaction deficits, stereotyped interests or behavior patterns, and difficulties adapting to change (5, 6). A prior research suggested that nonclinical ASD symptoms are found in the general adults population (7). In recent years, more and more scholars believed that autistic traits are continuously distributed across the general population (8–10). Many research indicated that individuals with ASD or high autistic traits are prone to have psychological problems such as anxiety, depression (6, 11–13). Some studies also found a close association between autistic traits and suicide (14–17). It has already been confirmed that autistic traits are related to the raising of suicide risk in both clinical and nonclinical population groups (15, 18–20). These results suggested that individuals with high autistic traits might be at particular danger of experiencing suicidal ideation. However, up to now, most published studies that examined the correlation between autistic traits and suicidal ideation have been carried out with small samples (11, 13). So it is necessary to explore it with large population-based samples.

Very few studies have explored the mechanisms underlying the relations between autistic traits and suicidal ideation. For example, alexithymia might be an influencing factor of suicidal ideation (21–24). However, whether it could reveal a greater danger of suicidal ideation or thoughts in individuals with high autistic traits has not been explored yet. Alexithymia, which indicates “no words for mood,” refers to difficulties in feeling, verbalizing, expressing and regulating emotions (25), and it has been traditionally treated as a stable personality trait rather than an independent psychiatric disorder (26). In recent years, studies have suggested individuals with ASD often have affective deficits, which are core symptoms of alexithymia (27–29). Alexithymia is more common among individuals with ASD compared to the general population. Individuals with ASD have increasing rates of alexithymia, with a prevalence of 40–65% reported in ASD individuals, versus 16% in typically-developing populations (30–32). Moreover, people with high autistic traits reported higher alexithymia scores (33, 34). Given that autistic traits are closely related to alexithymia and alexithymia has an impact on suicidal ideation, alexithymia might play a mediating role between autistic traits and suicidal ideation.

Furthermore, abnormal social reciprocity is the main clinical characteristics of ASD, including impairment of social communication, deficits of social imagination, and inability to engage in social interaction (35). Individuals with these characteristics might be at high risk of interpersonal relationship problems. Previous researches suggested that children with ASD are more often neglected and rejected by others, have difficulties initiating and maintaining friendships, have fewer playmates, are short of friendship with high quality, and feel more lonely than their generally developing peers (36, 37). These problems would persist into adulthood, bringing poor-quality relationships. Existing studies have reported that interpersonal problems might lead to mental health problems, especially suicidal ideation and behaviors (38–40). According to the interpersonal theory of suicide (41), suicidal ideation emerges when an individual experience interpersonal problems, such as feelings of perceived burdensomeness and thwarted belongingness. However, whether autistic traits could predict suicidal ideation through interpersonal problems has not been examined.

Based on our literature reviews, the present study aimed to address a gap in the literature in understanding how autistic traits affect suicidal ideation among large population-based nonclinical samples. For one thing, we examined the relationship between autistic traits and suicidal ideation. Specifically, this study hypothesized that autistic traits could affect suicidal ideation directly. For another, this study tested if autistic traits would affect suicidal ideation through the indirect effects of alexithymia and interpersonal problems. Specifically, we expected that alexithymia and interpersonal problems would play the mediating roles between autistic traits and suicidal ideation.

## 2 Methods

### 2.1 Subjects

A total of 6,973 Chinese college students were recruited from Zhengzhou(a developing city of central China). After eliminating the questionnaire with unfinished answers and obvious casually-answering patterns, there were 6,763 effective participants including 3,829 females, who were 16–35 years old, with the mean age of 21.00 (SD = 3.52). All participants in this research were voluntary.

### 2.2 Materials and measures

#### 2.2.1 Autism-Spectrum quotient

This scale is used to qualify autistic trait in all general populations (7). The Chinese version includes 50 items which cover five subscales: imagination, attention to detail, attention switching, communication, and social skills (42). After reading the items, the participants were asked to rate the degree to which each item one agree or disagree, on a 4-point Likert scale (ranging from definitely agree to definitely disagree). A high score denotes a great level of autistic traits. Participants whose scores are more than 32 can be classified as high autistic traits individuals. This scale was applied extensively among Chinese and reported good validity and reliability (42, 43). In this study, the Cronbach’s α coefficient of subscales ranged from 0.63 to 0.74, and the Cronbach’s α coefficient for the total score was 0.70.

#### 2.2.2 Toronto alexithymia scale-20

TAS-20 is designed to assess alexithymia in adults (44). It has been translated into Chinese version (45). This questionnaire is composed of 20 items and consists of three subscales: difficulty in identifying feelings, difficulty in describing feelings, and external oriented thinking. Each item is rated on a 5-point Likert scale ranging from “1 = strongly disagree” to “5 = strongly agree.” Total scores are between 20 and 100, with higher scores meaning higher degrees of alexithymia. Previous researches showed satisfactory validity and reliability (46, 47). Based on the current sample, the Cronbach’s α coefficient of subscales ranged from 0.79 to 0.83, and the Cronbach’s α coefficient for the total score was 0.82.

#### 2.2.3 Interpersonal problems

The interpersonal problems subscale was derived from The Chinese Adolescents Self-Rating Life Events Checklist to assess the extent to which people experienced interpersonal problems in the last 12 months (48). This subscale includes five items and each item is scored on a five-point scale from 1 (not at all) to 5 (extremely severe). If the event does not happen, it is counted as not at all. This scale was widely used and reported good validity and reliability in previous studies (49). The Cronbach’s α coefficient for this sample was 0.81.

#### 2.2.4 Suicidal ideation

Suicidal Ideation was measured by the items “wanting to end my life” and “thoughts about death,” from the revised Symptom Checklist (SCL-90-R) (50). These items can be answered on a 5-point scale (0 = not at all, 1 = a little bit, 2 = moderately, 3 = quite a bit, 4 = extremely). we calculated mean score of the two items as a suicidal ideation index. Participants whose scores were 0.73 or more were considered to have thoughts of committing suicide. Previous study has been successfully utilized these items to explore suicidal ideation (51).

### 2.3 Procedure

The Ethics Committee for Scientific Research of Zhengzhou University approved of the research. The survey was carried out online in mid-November 2019. The first page informed the participants that the research was entirely anonymous and voluntary, that data would be only used for scientific research, and that they could exit freely at any time. This research was developed in accordance with the 1989 revision of the Declaration of Helsinki.

### 2.4 Statistical analysis

Descriptive analysis and Zero-order Pearson’s correlations between variables were conducted by SPSS ver.21.0. PROCESS macro (model 6) was used to test the mediating effects of alexithymia and interpersonal problems (52, 53). Bias-corrected bootstrap estimates were based on 5,000 samples. If zero is not included in the 95% bootstrap confidence interval for the indirect effect, the mediating effect is significant.

## 3 Results

### 3.1 Common method bias test

The common method bias was tested by Harman’s single-factor test in this research (54). The results of exploratory factor analysis suggested the data were qualified (KMO = 0.94, Bartlett = 165459.47, *p* < 0.001), and the first factor under the unrotated condition accounted for 13.76% of the total variance (< 40%), indicating common method bias was insignificant.

### 3.2 Descriptive statistics and correlations

The proportion of participants with suicidal ideation was 9.54%, with 645 participants having suicidal ideation in total (suicidal ideation score ≥ 0.73). There were 104 (1.54%) high autistic traits participants (autistic traits score > 32).

Table 1 shows descriptive statistics of each measure and correlations between them. The results of correlation analysis indicated that suicidal ideation was positively correlated with autistic traits, alexithymia and interpersonal problems (*r* = 0.17, *p* < 0.01; *r* = 0.31, *p* < 0.01; *r* = 0.32, *p* < 0.01). Similarly, higher scores on interpersonal problems were correlated with higher autistic traits and alexithymia (*r* = 0.23, *p* < 0.01; *r* = 0.40, *p* < 0.01). Finally, autistic traits and alexithymia were positively correlated (*r* = 0.38, *p* < 0.01).

**Table 1:** Descriptive statistics and correlations between variables.

Variables	*M*	*SD*	1	2	3	4	5
1. Age	21.00	3.52	1				
2. Gender	1.57	0.50	0.10^**^	1			
3. Autistic traits	21.43	5.42	−0.11^**^	−0.07^**^	1		
4. Alexithymia	2.51	0.55	−0.23^**^	−0.02	0.38^**^	1	
5. Interpersonal Problems	7.88	2.90	−0.18^**^	−0.03^**^	0.23^**^	0.40^**^	1
6. Suicidal Ideation	0.19	0.46	−0.15^**^	−0.02^**^	0.17^**^	0.31^**^	0.32^**^

^*^p < 0.05, ^**^p < 0.01, ^***^p < 0.001. 1 = male, 2 = female.

### 3.3 Mediation analysis

**Figure 1:**
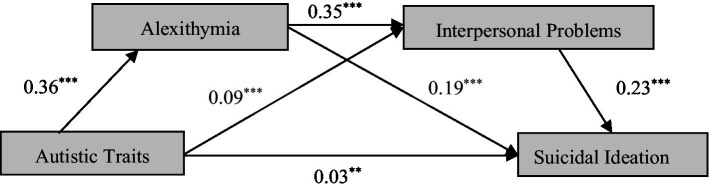
The standardized regression coefficient of each path. Model covariates include gender and age. ^*^p < 0.05, ^**^p < 0.01, ^***^p < 0.001.

PROCESS macro (model 6) was used to test the mediating roles of alexithymia and interpersonal problems between autistic traits and suicidal ideation. After converting all the scores to z-scores and controlling the effects of age and gender, the regression coefficients of each path were statistically significant (see Figure 1).

Besides, bootstrap estimates were calculated with 5,000 bootstrap samples. The results (see Table 2) suggested that alexithymia played a mediating role on the relationship between autistic traits and suicidal ideation (indirect effect = 0.068, 95% CI [0.056, 0.078], the ratio of indirect effect to total effect = 44.94%). And interpersonal problems’s mediating effect was also statistically significant (indirect effect = 0.021, 95% CI [0.015, 0.029], the ratio of indirect effect to total effect = 14.12%). Besides, there existed sequential mediating effect of alexithymia and interpersonal problems (indirect effect = 0.029, 95% CI [0.024, 0.035], the ratio of indirect effect to total effect = 19.04%). That means autistic traits not only directly affect suicidal ideation, but also indirectly increase suicidal ideation through the mediating effects of alexithymia and interpersonal problems.

**Table 2:** Mediating effects of the model.

Model	Ratio (%)	Effect	SE	Boot LLCI	Boot ULCI
Total Indirect Path	78.10	0.117	0.008	0.103	0.133
Ind 1. Autistic Traits → Alexithymia → Suicidal Ideation	44.94	0.068	0.006	0.056	0.078
Ind 2. Autistic Traits → Alexithymia → Interpersonal Problems → Suicidal Ideation	19.04	0.029	0.003	0.024	0.035
Ind 3. Autistic Traits → Interpersonal Problems → Suicidal Ideation	14.12	0.021	0.003	0.015	0.029

Ratio (%), the ratio of indirect effect to total effect; Boot LLCI, Bootstrap lower limit of confidence interval; Boot ULCI, Bootstrap upper limit of confidence interval.

## 4 Discussion

The present research sought to expand previous studies by examining the associations between autistic traits and suicidal ideation, and testing the mediating roles of alexithymia and interpersonal problems among large nonclinical samples. In accordance with prior findings, autistic traits were positively associated with suicidal ideation (19, 55–57). Additionally, in line with our hypothesis, the mediating effects of alexithymia and interpersonal problems on the correlation between autistic traits and suicidal ideation were significant. On the one hand, autistic traits were positively related to alexithymia, which influenced suicidal ideation in turn. Autistic traits also predict suicidal ideation through interpersonal problems. On the other hand, the effect of autistic traits on suicidal ideation was sequentially mediated by alexithymia and interpersonal problems.

The results indicated individuals with a high level of autistic traits are more prone to have suicidal ideation. Several prior studies have supported the fact that suicidal thoughts or behaviors are more prevailing among autistic individuals than the general people (14, 58). Firstly, autistic individuals are likely to have mental health problems like anxiety and depressive symptoms (11, 59), which are important risk factors for suicide. Secondly, social deficits and difficulties in initiating and maintaining interpersonal relationships are triggers for suicide in autistic individuals (55).

In addition, our findings further uncovered the mediating role of alexithymia in the association between autistic traits and suicidal ideation. As expected, autistic traits are positively associated with alexithymia. These results have been confirmed by previous studies (30, 33). It has been reported that alexithymia has a high rate of co-occurrence with autism in young people with ASD (31). Moreover, because of difficulties in expressing feelings effectively and understanding the emotional responses of others (25), individuals with high alexithymia may have not only interpersonal relationship problems but suicidal ideation (60, 61). For individuals with a high level of autistic traits who already have communication difficulties and social deficits, alexithymia that makes it harder to identify and communicate emotional feeling may increase their risk of suicidal ideation.

Our findings showed that interpersonal problems is an critical predictor of suicidal ideation. According to the interpersonal theory of suicide, thwarted belong and perceived cumbersomeness are two interpersonal factor that might lead to suicidal thoughts or behaviors (41). Autistic individuals lack social and communication skills, which lead to difficulties establishing and maintaining social relationships and support networks (19). They even experience social exclusion, isolation, and loneliness (18), and these may lead to mental health problems and suicidal ideation. Therefore, interpersonal problems can exacerbate the suicidal ideation of people with autistic traits.

### 4.1 Limitations

Several limitations need to be acknowledged in our study. First and foremost, the cross-sectional nature of the design fundamentally limits the ability to draw causal inferences (62). Although mediation analyses were conducted to explore potential pathways, the correlational nature of the data means that the direction of relationships and the mechanisms underlying them remain speculative. Longitudinal or experimental studies are necessary to confirm the temporal precedence and causality suggested by the current results. Secondly, the assessment of suicidal ideation relied solely on two items from the SCL-90-R. Although these items are widely used and have been validated in previous studies—including in large-scale surveys—this approach may not capture the full complexity of suicidal ideation, such as variations in intensity, planning, or transient risk. More comprehensive and clinically validated instruments, such as the Beck Scale for Suicide Ideation or the Columbia-Suicide Severity Rating Scale, are recommended for future research to provide a more nuanced and reliable measurement. Third, the current study failed to control the some covariates, including depressive symptoms, anxiety, prior suicide attempts, traumatic experiences, family psychiatric history, and other mental health-related factors. These unmeasured variables may serve as potential confounders and could influence the observed associations between autistic traits, alexithymia, interpersonal problems, and suicidal ideation. It is possible that the effects attributed to these constructs may be partially mediated or explained by these omitted variables. Future studies should systematically assess and control for these important confounding factors to enhance the validity and interpretability of the findings. In addition, the generalizability of our findings is limited by the specific characteristics of our sample, which consisted exclusively of highly educated college students from a single geographic region. This restricts the extrapolation of the results to broader populations with diverse educational, socioeconomic, or cultural backgrounds. Future studies should seek to include more heterogeneous samples—such as individuals from varying education levels, occupational contexts, and regional settings—to enhance the external validity and broader applicability of the findings. Lastly, due to embarrassment, shame, and social desirability, there may exist bias in the data. So the anonymity of questionnaires is a recommended way to reduce it.

### 4.2 Implications

The findings of the present study carry several practical implications, particularly for the development and enhancement of mental health interventions within university settings. First, the identified mediating role of alexithymia suggests that incorporating routine screening for alexithymia in college mental health assessments may help identify students at heightened risk of suicidal ideation, especially those with elevated autistic traits. Implementing such screenings could facilitate early detection and intervention. Second, given the significant mediating effect of interpersonal problems, integrating social skills training into campus counselling programs or peer-support initiatives may be beneficial. These programs could focus on enhancing emotional communication, building interpersonal connections, and reducing feelings of isolation—core factors addressed in our mediation model. Finally, the results underscore the need for tailored counselling approaches that simultaneously target emotion recognition and expression (addressing alexithymia) and interpersonal effectiveness. Developing intervention modules that combine psychoeducation, cognitive-behavioural techniques, and group-based activities may provide a comprehensive support mechanism for students with high autistic traits, thereby potentially mitigating suicide risk.

## 5 Conclusion

In conclusion, although previous research suggests that a high level of autistic traits is related to more suicidal thoughts and behaviors, the specific underlying mechanism is not clear. The present study expands prior findings, confirms the direct relationship between autistic traits and suicidal ideation, and further illuminates the mediating roles of alexithymia and interpersonal problems in this relationship among large population-based college samples. The findings contribute to our further understanding of the relationship between autistic traits and suicidal ideation in the context of other complex risks and outcomes. Besides, future research is needed to explore if reducing alexithymia and interpersonal problems might be helpful to the prevention and treatment of suicidal ideation.

## Data Availability

The raw data supporting the conclusions of this article will be made available by the authors, without undue reservation.
